# Isokinetic force-power profile of the shoulder joint in males participating in CrossFit training and competing at different levels

**DOI:** 10.7717/peerj.11643

**Published:** 2021-09-17

**Authors:** Maximiliano A. Torres-Banduc, Daniel Jerez-Mayorga, Jason Moran, Justin W.L. Keogh, Rodrigo Ramírez-Campillo

**Affiliations:** 1 Escuela de Kinesiología, Facultad de Ciencias de la Salud, Universidad de Las Americas, Viña del Mar, Chile; 2 Escuela de Ciencias de la Salud, Universidad de Viña del Mar, Viña del Mar, Chile; 3 Department Physical Education and Sports. Faculty of Sport Sciences, University of Granada, Granada, Spain; 4 Facultad de Ciencias de la Rehabilitación, Universidad Andres Bello, Santiago, Chile; 5 School of Sport, Rehabilitation and Exercise Sciences, University of Essex, Essex, United Kingdom; 6 Faculty of Health Sciences and Medicine, Bond University, Gold Coast, Australia; 7 Sports Performance Research Centre New Zealand, AUT University, Auckland, New Zealand; 8 Cluster for Health Improvement, Faculty of Science, Health, Education and Engineering, University of the Sunshine Coast, Sunshine Coast, Australia; 9 Kasturba Medical College, Manipal Academy of Higher Education, Manipal, India; 10 Department of Physical Activity Sciences, Universidad de Los Lagos, Osorno, Chile

**Keywords:** Injuries, Strength training, Resistance training, Human physical conditioning, Sports, Plyometric exercise, Athletic performance, Physical education and training, Exercise therapy, Return to sport

## Abstract

**Background:**

As participants who engage in CrossFit training and competition perform a large volume of high intensity overhead activities, injuries to the shoulder are one of the most common in this sport. Previous research in other sports has indicated that the isokinetic force power profile of the shoulder joint (IPSJ) rotator muscles may assist in the prediction of shoulder injury.

**Aim:**

Therefore, the objective of this study was to determine the IPSJ in males engaged in CrossFit training at different competitive levels.

**Methods:**

In a cross-sectional study design, participants (age, 24.1 ± 2.7 years) classified as ‘beginner’ (*n* = 6), ‘intermediate’ (*n* = 7) or ‘advanced’ (*n* = 9) provided informed consent to participate in this study. The IPSJ assessment involved rotational and diagonal movements, including internal and external shoulder rotator muscles, at both 180°.s^−1^ and 300°.s^−1^. The variables analysed were peak torque/body mass (%), mean power (W) and the external/internal peak torque/body mass ratio (%). A Kruskal–Wallis test was used to compare the IPSJ of the three groups, with Dunn’s test used for post-hoc analysis. The alpha level was set at *p* < 0.05.

**Results:**

The IPSJ showed greater torque and power values in those who competed at the advanced level as compared to those at a lower competitive level (*i.e*. intermediate, beginner). This was observed mainly for the internal rotation and internal diagonal movements at both 180°.s^−1^ and 300°.s^−1^. However, such differences between competitive levels were, in general, absent for the external rotation and external diagonal movements. Moreover, the participants from the advanced competitive level exhibited an imbalance of peak torque between the muscles responsible for the external–internal rotational and external-internal diagonal movements of the shoulder (*i.e*. peak torque external/internal ratio <66%), particularly in the dominant shoulder.

**Conclusion:**

These findings suggest greater development of the shoulder internal rotators and a higher probability of shoulder injury in CrossFit athletes at the advanced competitive level. Based on these results, participants engaged in CrossFit training and competition may wish to increase the volume of training for the shoulder external rotator muscles to complement the large increases in shoulder internal rotator strength that occur as a part of their regular training regimes.

## Introduction

CrossFit is practiced in 142 countries worldwide (Claudino et al., 2018) and involves participants competing in a range of diverse athletic events ranging from those requiring short-duration maximal efforts (*e.g*. one repetition maximal barbell lifting) to longer-duration, lower-intensity efforts (*e.g*. endurance-dominant events), with some events offering information on the nature of competition only a few moments prior to participation (Pritchard, Keogh & Winwood, 2020). CrossFit training usually involves several high-intensity functional movements, including mono-structural (*e.g*., cardiovascular activities such as running and rowing), body-weight (*e.g*., push-ups; derived from gymnastics) and weightlifting derivatives (*e.g*., snatch, shoulder press, deadlift), executed quickly, repetitively, and with little or no recovery time between sets (Claudino et al., 2018; Feito et al., 2018; Schlegel, 2020). Competitors engaged in CrossFit may train ~11 h per week (Pritchard, Keogh & Winwood, 2020), with greater volumes of activity being observed in higher level participants.

Several studies report that the shoulder joint is the most affected by overuse injuries in Crossfit practitioners (Klimek et al., 2018), particularly in males (Dominski et al., 2018; Weisenthal et al., 2014). This finding is supported by the results of a systematic review of weight training sports, such as weightlifting, strongman and powerlifting, in which the shoulder is typically the most frequently injured body part due to the execution of heavily loaded overhead exercises (Keogh & Winwood, 2017). It is also indicated in a recent study (Carbone, Candela & Gumina, 2020) that the type of exercises followed by CrossFit participants (*i.e*. activities that involve abduction and forward elevation with external rotation under load-bearing) could lead to a lesion in the active stabilizers of the aforementioned joint. As the mechanisms associated with shoulder injuries in this sport are still not entirely clear, it is now necessary to identify the possible causes and factors connected with shoulder injuries in CrossFit athletes with a view to reducing injury rates, (Da Costa et al., 2019).

Assessments of isokinetic force-power profiles have been used in different sports to identify the factors associated with injuries in different joints (Alt, Knicker & Strueder, 2017; Prill et al., 2019; Siddle et al., 2018), including the shoulder (Ellenbecker & Davies, 2000). Indeed, the isokinetic force-power profile of the shoulder joint (IPSJ) may be used to predict the risk of injury using i) the relationship of peak torque between agonist/antagonist muscles , ii) peak torque in relation to body mass (peak torque/body mass), iii) muscle power, and/or iv) symmetry indexes between limbs (Alt, Knicker & Strueder, 2017; De la Motte et al., 2017; Motta et al., 2018; Prill et al., 2019; Tasiopoulos et al., 2018; Vanderstukken et al., 2019). This could be particularly applicable in sports such as volleyball (Mendonça et al., 2010), hockey (Vanderstukken et al., 2019), basketball (Weissland et al., 2018), handball (Van Cingel et al., 2018), boxing (Tasiopoulos et al., 2018), and other sports that require participants to execute a large volume of high intensity shoulder movements (Motta et al., 2018), such as in CrossFit. In addition to its potential to determine the risk of injury, the IPSJ could facilitate the establishment of threshold values for return to sports practice after a shoulder injury (Ellenbecker & Davies, 2000).

Based on the shoulder typically being the most frequently injured body part in CrossFit, it is somewhat surprising that the IPSJ has not been determined in CrossFit participants thus far. It is also unclear as to how CrossFit participants who compete at different levels (*e.g*. competitive; recreational) may differ in their IPSJ profile and risk of shoulder injury (Mangine et al., 2020). Indeed, recent studies suggest that those CrossFit participants who have competed in official events have more experience in CrossFit training and/or training under supervision, meaning the incidence of shoulder injuries is reduced (Feito et al., 2020; Klimek et al., 2018). Accordingly, the objective of this study was to determine the IPSJ in male CrossFit participants of different competitive levels. Based on previous studies of the IPSJ in other sports (Mendonça et al., 2010; Miller et al., 2018; Tasiopoulos et al., 2018), we hypothesised that because of the large volume of training of the internal rotator muscles in key CrossFit movements, advanced level participants would have greater internal rotator strength and power but that the external to internal shoulder rotator ratios may be compromised.

## Methods

In a cross-sectional study design, male CrossFit training participants from three different competitive levels (advanced, intermediate, and beginner) were assessed for IPSJ.

### Participants

Twenty-two male CrossFit participants were included in this study (basic descriptive characteristics in Table 1). No significant differences were observed for the variables of age (*p* = 0.512), height (*p* = 0.918) or body mass (*p* = 0.426) between the three groups of participants.

**Table 1:** Basic descriptive characteristics of male CrossFit athletes according to their competitive level.

	Advanced (*n* = 9)	Intermediate (*n* = 7)	Beginner (*n* = 6)
Age (years)	24.4 ± 1.3 (23.0–26.0)	23.4 ± 3.6 (18.0–29.0)	24.3 ± 3.6 (21.0–31.0)
Height (m)	1.73 ± 0.07 (1.64–1.83)	1.73 ± 0.04 (1.69–1.80)	1.72 ± 0.07 (1.63–1.83)
Body mass (kg)	84.56 ± 9.2 (69.0–94.0)	78.71 ± 9.9 (67.0–99.0)	78.3 ± 12.7 (65.0–96.0)

**Note:**

Values are mean ± standard deviation (minimum and maximum values). No significant differences between groups.

The participants were selected based on the following inclusion criteria: (i) self-reported experience in CrossFit training of ≥36 months of regular training (≥3 sessions of ≥120 min per week) for the advanced experienced level group; 12 to <36 months for intermediate competitive level; 6 to <12 months for the beginner competitive level; (ii) no injury sustained in the three months prior to the study. The exclusion criteria were: (i) musculoskeletal problems affecting the ability to exert muscle strength and/or participate in the isokinetic evaluation of the shoulder; (ii) self-reported use of anabolic steroids or another prohibited substance that could affect the results of the study.

Data for this study arose as a condition of employment whereby athletes’ sport clubs paid for their athletes to be assessed. However, the study was carried out in accordance with the recommendations of the latest version of the Helsinki declaration (WMA, 2013) and the participants were informed about the procedures, risks and benefits associated with the isokinetic assessment in an ethical manner. Written informed consent was obtained from all participants before any data collection was performed. In addition, the director of the University of Las Americas movement analysis laboratory, where the assessments took place, authorised access to the isokinetic equipment database. Moreover, legal regulations were followed, according to national law (19.628, *i.e*. privacy and protection of personal data), where assessments were performed. The University of Las Americas granted ethical approval to carry out the study within its facilities (ethical application ref: CEC_FP_2020029).

### Procedures

Before measurements, the participants completed a 15-min familiarisation session in which the isokinetic tests were explained and a practice trial for each was performed. The participants were instructed to avoid vigorous physical training for 48 h before the testing session and to maintain their usual diet during this period. The same research assistants (unaware of the study aim and the Crossfit competition level of the participants) carried out the familiarisation and testing sessions, in the same laboratory, under controlled environmental conditions, between 10:00 and 11:00 a.m.

#### Anthropometric evaluation

Body mass was measured using a calibrated mechanical scale (SECA, model 711, Hamburg, Germany), with a precision of 0.1 kg. Standing height was measured using a telescopic scale (SECA, model 220, Hamburg, Germany), with a precision of 0.1cm.

#### Isokinetic evaluation

**Figure 1:**
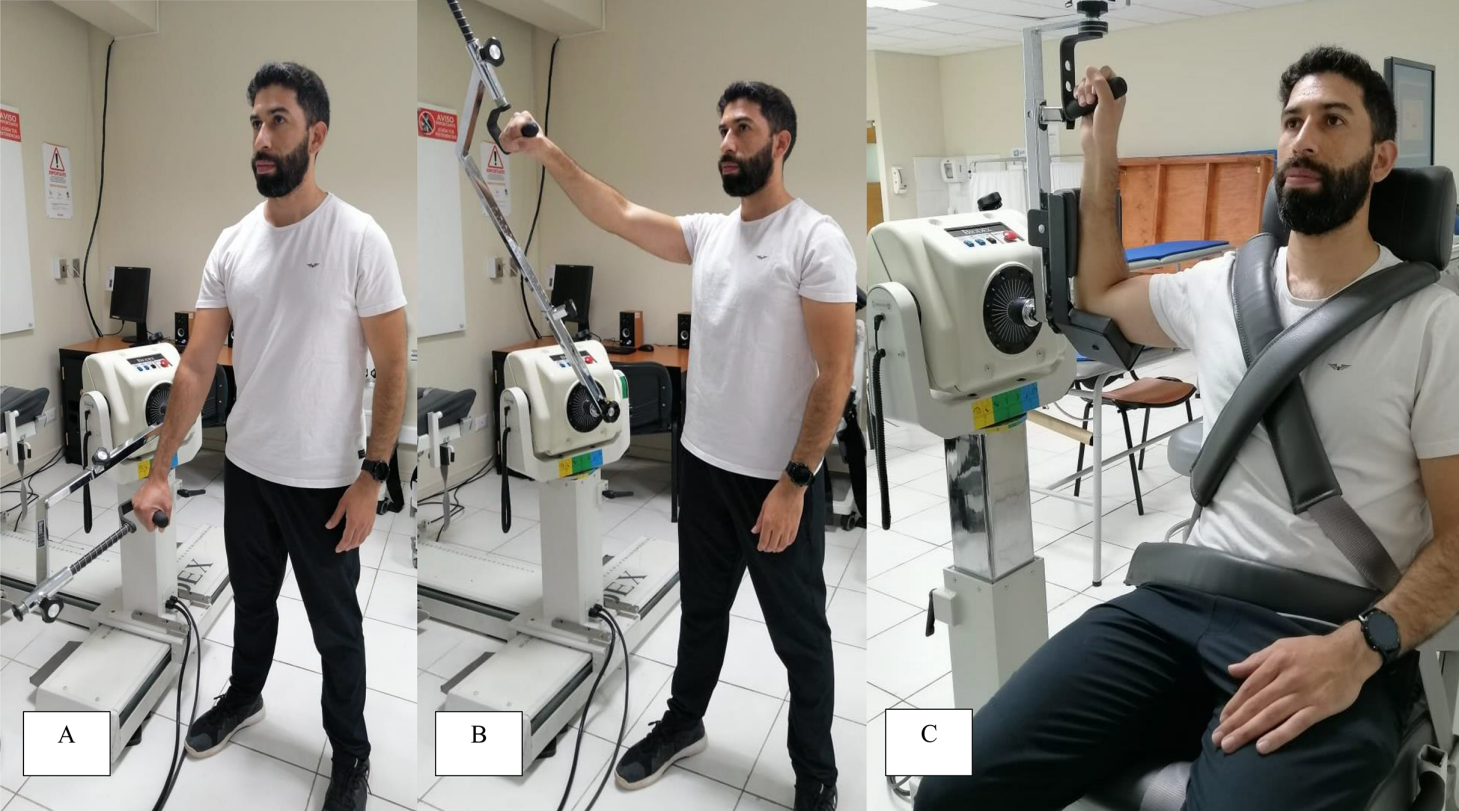
Measurement of the isokinetic force-power profile of the shoulder joint in a representative male CrossFit athlete. (A) and (B) External diagonal movement. The inverse pattern of movement allowed the measurement of internal diagonal parameters. (C) Initial position for the measurement of internal-external rotational performance.

As there are no established protocols for CrossFit at present, according to biomechanical evaluations of the shoulder joint and expert judgment, two movements were selected to assess the IPSJ in the participants: rotational and diagonal movements (Fig. 1). For both the rotational and diagonal movements, internal and external shoulder muscle activation was required, consistent with muscles’ role in inducing movement and providing stability around the shoulder joint (Weissland et al., 2018). All movement patterns were measured at 180°.s^−1^ and 300°.s^−1^. Different movement speeds were selected since they differently represent strength imbalances and injury risk (Kabacinski et al., 2018; Rosene, Fogarty & Mahaffey, 2001). In addition, the speeds were selected considering the high velocity shoulder movements that CrossFit participants typically perform during training and competition (Claudino et al., 2018; Mangine et al., 2020; Pritchard, Keogh & Winwood, 2020). Moreover, torque and power were measured across movements and speeds, since both torque and power are considered independent markers of the training level of athletes (Toskić et al., 2020), with such data perhaps being useful to discriminate between different competitive levels of athletes within the same sport.

The isokinetic measurements were performed using valid and reliable (Drouin et al., 2004) equipment (Biodex^®^, System 3 Pro, NY, USA) with a precision of ±1%. Before maximal measurements, a 5-min warm-up was performed, including shoulder movements in different directions, with a focus on internal and external rotation and internal and external diagonal movements, with a progression up to throwing medicine balls of different masses in different directions. In addition, the warm-up also comprised of low-intensity dynamic stretching (Opplert & Babault, 2018) and three submaximal repetitions of the isokinetic movements. After 30 s of rest, each participant performed a set of five maximal concentric repetitions at each speed for each movement pattern. The full measurement protocol (*i.e*. both limbs) involved 16 sets of five repetitions, with a 2-min rest between sets. One limb was measured first before the measurement of the contralateral limb. The order of limb measurement was selected randomly (*i.e*. bag method). To determine limb dominance, the participant was asked which hand they use to write with and this information was confirmed during the signing of the informed consent form. During measurement, the participants were encouraged to exert maximum effort using a standardised protocol.

For the rotational movements, the shoulder joint was set at 90° of abduction in the frontal plane, considering an arc of movement of approximately 105° (Weissland et al., 2018). The dynamometer was set with 5° of inclination and without rotation (0°). The participant was seated on the dynamometer chair and the shoulder was stabilized with straps added at the thigh and hip level to minimise the movement of the trunk and other limbs. The shoulder/elbow accessory of the measurement equipment was then put in place and the dynamometer was raised to align its axis with the axis of rotation of the humerus. Once secured and with the segment stabilised, the previously described testing protocol (*i.e*. warm-up and maximal effort) was performed. Afterwards, the same procedure was carried out on the contralateral limb.

For the diagonal measurements, the dynamometer was tilted 30° and rotated to 35°. The participant was asked to maintain a bipedal position perpendicular to the axis of the dynamometer, with the shoulder/elbow accessory of the dynamometer adjusted to the length of the upper limb, as previously outlined (Alonso-Cortés et al., 2006). Once these adjustments were made, the previously described testing protocol (*i.e*. warm-up and maximal effort) was performed on both limbs.

For the rotational and diagonal movements, the variables analysed were peak torque/body mass (*i.e*. maximum torque relative to total body mass (%)), mean power (W), and the external/internal ratio (%), which is calculated as:

$${\rm External/Internal\; ratio\; (\% )\; = \; }\displaystyle{{{\rm Peak\; external\; torque}} \over {{\rm Peak\; internal\; torque}}}\times{100}$$

### Statistical analysis

To calculate the sample size, statistical software (G*Power, v3.1.9.7, Heinrich-Heine-Universität, Germany) was used. Given the study design (three independent groups, one measure), a large effect size = 0.9, obtained from a partial eta squared of 0.485 (Mangine et al., 2020), was used. Considering the above, and a desired power (1-ß error) = 0.8, alpha-error < 0.05, the total sample size was 18 participants (*i.e*. 6 per group). Considering potential attrition, the minimal initial sample size was set at 22 participants.

Data normality was assessed using the Shapiro–Wilk test. As the majority of data were non-normally distributed, most are presented as medians and interquartile ranges. However, as age, body mass and height were normally distributed they have been presented as means and standard deviations. Only data with a coefficient of variation ≤10% (obtained from five repetitions per test) were used in the analyses (Motta et al., 2018). Due to the non-normality of the data, the Kruskal–Wallis test was used to determine if there were significant differences between groups for the IPSJ. The eta squared (η^2^) value is provided as a measure of the effect size. The alpha level was set at *p* < 0.05. Where significant differences were found, a *post hoc* analysis was performed using Dunn’s test. Statistical analyses were performed for each limb separately. All calculations were performed using Prism 5 software (GraphPad Software, California, USA).

## Results

The results for rotational and diagonal peak torque relative to body mass are shown in Tables 2 and 3, respectively. The advanced competition level participants had significantly greater internal rotation peak torque relative to body mass at 180°.s^−1^ than the beginner participants (*p* = 0.015, η^2^ = 0.334, dominant limb) and at 300°.s^−1^ than the intermediate participants (*p* = 0.010, η^2^ = 0.377, dominant limb; *p* = 0.005, η^2^ = 0.441, non-dominant limb) (Table 2). Similarly, internal diagonal peak torque relative to body mass at 180°.s^−1^ (*p* = 0.038, η^2^ = 0.239, dominant limb) and 300°.s^−1^ (*p* = 0.010, η^2^ = 0.376, dominant limb) was significantly higher in the advanced compared to intermediate level participants (Table 3).

**Table 2:** Rotational peak torque relative (%) to body mass in dominant and non-dominant shoulder.

	Advanced (*n* = 9)		Intermediate (*n* = 7)		Beginner (*n* = 6)		Dominant^£^		Non-dominant^£^	
	Dominant	Non-dominant	Dominant	Non-dominant	Dominant	Non-dominant	p	η^2^	p	η^2^
External rotation 180°.s^−1^	42.5 (33.9–48.5)	39.1 (36.8–47.8)	41.4 (37.5–50.1)	40.0 (37.6–42.1)	37.2 (28.9–45.4)	38.5 (26.5–51.8)	0.296	0.023	0.754	0.076
External rotation 300°.s^−1^	43.8 (40.9–47.2)	40.9 (39.0–47.9)	42.9 (38.6–43.8)	42.3 (35.8–45.4)	40.6 (34.6–50.5)	42.5 (35.7–46.2)	0.446	0.02	0.895	0.094
Internal rotation 180°.s^−1^	72.2 (59.8–86.9)	70.5 (62.8–78)	54.8 (51.8–60.0)	58.9 (49.9–66.2)	48.3 (43.8–66.5)*	59.3 (49.2–74.9)	0.015	0.334	0.075	0.168
Internal rotation 300°.s^−1^	77.2 (65.5–84.9)	71.0 (61.2–86)	51.6 (45.4–58.7)*	47.8 (35.3–59.2)*	59.4 (50.9–72.8)	57.4 (51.4–66.9)	0.010	0.377	0.005	0.441

**Notes:**

* Beginner (*p* < 0.05 compared to Advanced). Values are median (interquartile range).

£ The *p* and η^2^ values were derived from Kruskal–Wallis between-groups comparison.

**Table 3:** Diagonal peak torque relative (%) to body mass in dominant and non-dominant shoulder.

	Advanced (*n* = 9)		Intermediate (*n* = 7)		Beginner (*n* = 6)		Dominant^£^		Non-dominant^£^	
	Dominant	Non-dominant	Dominant	Non-dominant	Dominant	Non-dominant	p	η^2^	p	η^2^
External diagonal 180°.s^−1^	64.2 (50.6–97.4)	82.2 (59.3–90.8)	51.8 (45.7–64.9)	51.3 (44.8–62.3)	58.6 (45.5–63.3)	67.6 (56.7–75.5)	0.603	0.052	0.079	0.161
External diagonal 300°.s^−1^	74.3 (51.5–93.9)	80.1 (50.8–83.7)	47.7 (44.3–70.1)	52.6 (46.1–62.3)	56.5 (47.0–71.1)	65.3 (56.8–72.2)	0.334	0.01	0.275	0.031
Internal diagonal 180°.s^−1^	118.5 (94.8–125.1)	119.8 (90.5–126.4)	72.7 (62.3–106.6)*	73.1 (64.3–102.1)	98.2 (77.4–105.4)	97.5 (87.3–104.3)	0.038	0.239	0.094	0.143
Internal diagonal 300°.s^−1^	111.3 (95.1–142.3)	109.4 (81.5–119.2)	60.5 (45.3–90.1)*	63.5 (44.0–94.6)	81.2 (69.1–104.1)	92.7 (79.4–108.6)	0.010	0.376	0.054	0.201

**Notes:**

* Beginner (p<0.05 compared to Advanced). Values are median (interquartile range).

£ The p and η^2^ values were derived from Kruskal–Wallis between-groups comparison.

The results for rotational and diagonal mean power are shown in Tables 4 and 5, respectively. The internal rotation mean power at 180°.s^−1^ was greater in the advanced level compared to intermediate (*p* = 0.006, η^2^ = 0.424, non-dominant limb) and beginner (*p* = 0.008, η^2^ = 0.400, dominant limb) participants (Table 4). In addition, the internal rotation mean power at 300°.s^−1^ was greater in the advanced level compared to intermediate (*p* = 0.002, η^2^ = 0.523, dominant limb; *p* < 0.001, η^2^ = 0.646, non-dominant limb) and beginner (*p* = 0.016, dominant limb; *p* = 0.017, non-dominant limb) participants (Table 4). Moreover, the internal diagonal mean power at 180°.s^−1^ was greater in the advanced level compared to the intermediate (*p* = 0.003, η^2^ = 0.483, dominant limb; *p* = 0.003, η^2^ = 0.499, non-dominant limb) and beginner (*p* = 0.029, dominant limb) (Table 5) participants. In addition, the internal diagonal mean power at 300°.s^−1^ was greater in the advanced level compared to the intermediate (*p* = 0.006, η^2^ = 0.434, dominant limb; *p* = 0.025, η^2^ = 0.281, non-dominant limb) (Table 5) participants. External diagonal mean power at 300°.s^−1^ was greater in the advanced level compared to the intermediate level (*p* = 0.022, η^2^ = 0.293, non-dominant limb) (Table 5) participants.

**Table 4:** Rotational mean power (W) in dominant and non-dominant shoulder.

	Advanced (*n* = 9)		Intermediate (*n* = 7)		Beginner (*n* = 6)		Dominant^£^		Non-dominant^£^	
	Dominant	Non-dominant	Dominant	Non-dominant	Dominant	Non-dominant	p	η^2^	p	η^2^
External rotation 180°.s^−1^	65.1 (55.6–70.9)	60.5 (51.6–70.7)	54.5 (51.3–90.5)	54.8 (42.7–65.3)	48.2 (29.8–58.3)	55.9 (26.3–63.0)	0.069	0.176	0.456	0.023
External rotation 300°.s^−1^	78.4 (59.6–91)	72.6 (64.6–85.9)	61.3 (60.5–72.2)	60.7 (54.6–66.7)	69.9 (49.1–76.0)	69.8 (54.0–79.8)	0.391	0.006	0.052	0.206
Internal rotation 180°.s^−1^	98.5 (82.0–136.7)	96.2 (91.6–127.1)	75.7 (68.1–91.5)	76.8 (72.4–81.0)*	62.4 (31.9–85.1)*	77.8 (48.5–98.4)	0.008	0.400	0.006	0.424
Internal rotation 300°.s^−1^	148.2 (111.2–160.9)	125.6 (116.1–154.4)	79.6 (66.2–93.6)*	77.8 (66.6–85.3)*	84.3 (64.7–107.5)*	96.2 (57.4–110.7)*	0.002	0.523	<0.001	0.646

**Notes:**

* Beginner (*p* < 0.05 compared to Advanced). Values are median (interquartile range).

£ The p and η^2^ values were derived from Kruskal–Wallis between-groups comparison.

**Table 5:** Diagonal mean power (W) in dominant and non-dominant shoulder.

	Advanced (*n* = 9)		Intermediate (*n* = 7)		Beginner (*n* = 6)		Dominant^£^		Non-dominant^£^	
	Dominant	Non-dominant	Dominant	Non-dominant	Dominant	Non-dominant	p	η^2^	p	η^2^
External diagonal 180°.s^−1^	74.0 (67.6–116.5)	85.4 (72.6–121.0)	72.7 (57.1–123.0)	66.6 (56.1–70.4)	60.9 (45.2–103.5)	70.9 (60.7–101.6)	0.430	0.016	0.072	0.171
External diagonal 300°.s^−1^	85.7 (72.6–133.7)	94.8 (85.3–159.2)	69.2 (47.4–108.1)	70.6 (54.6–72.7)*	64.8 (48.9–96.7)	83.4 (63.8–92.3)	0.126	0.113	0.022	0.293
Internal diagonal 180°.s^−1^	161.4 (147.1–203.3)	170.1 (146.7–215.4)	96.3 (70.3–136.8)*	96.3 (80.6–130.6)*	120.1 (108.8–127.0)*	134.9 (123.2–137.9)	0.003	0.483	0.003	0.499
Internal diagonal 300°.s^−1^	189.4 (162.2–274.5)	196.9 (155.8–256.3)	80.5 (66.0–175.0)*	103.4 (75.2–173.1)*	132.8 (124.6–151.1)	136.1 (126.6–174.8)	0.006	0.434	0.025	0.281

**Notes:**

* Beginner (*p* < 0.05 compared to Advanced). Values are median (interquartile range).

£ The p and η^2^ values were derived from Kruskal–Wallis between-groups comparison.

The external/internal ratio for peak torque relative to body mass in the dominant and non-dominant shoulders are indicated in Table 6, for both rotational and diagonal movements. A lower rotational external-internal ratio at 180°.s^−1^ (*p* = 0.014, η^2^ = 0.346, dominant limb) and 300°.s^−1^ (*p* = 0.025, η^2^ = 0.282 dominant limb) was noted in the advanced level compared to the intermediate level participants.

**Table 6:** External/internal ratio for peak torque (%) in dominant and non-dominant shoulder.

	Advanced (*n* = 9)		Intermediate (*n* = 7)		Beginner (*n* = 6)		Dominant^£^		Non-dominant^£^	
	Dominant	Non-dominant	Dominant	Non-dominant	Dominant	Non-dominant	p	η^2^	p	η^2^
Rotational external-internal ratio 180°.s^−1^	57.4 (53.9–71.5)	59.0 (52.3–65.1)	72.8 (69.5–75.6)*	67.9 (56.5–84.3)	70.3 (65.9–76.9)	62.8 (53.8–71.7)	0.014	0.346	0.181	0.075
Rotational external-internal ratio 300°.s^−1^	56.6 (54.2–65.9)	61.6 (50.1–67.1)	76.4 (70.1–79.6)*	82.8 (58.0–88.5)	66.4 (61.1–81.2)	70.2 (63.2–73.3)	0.025	0.282	0.036	0.245
Diagonal external-internal ratio 180°.s^−1^	60.8 (51.6–72.3)	61.1 (49.9–76.4)	71.3 (46.1–115.7)	55.4 (50.7–68.0)	59.1 (56.4–62.3)	70.3 (57.5–77.4)	0.799	0.082	0.596	0.051
Diagonal external-internal ratio 300°.s^−1^	67.5 (51.5–70.9)	68.5 (51.7–75.5)	72.6 (64.4–106.3)	77.4 (62.2–99.0)	74.4 (62.6–84.8)	66.8 (54.8–95.4)	0.118	0.119	0.587	0.049

**Notes:**

* Beginner (*p* < 0.05 compared to Advanced). Values are median (interquartile range).

£ The *p* and η^2^ values were derived from Kruskal–Wallis between-groups comparison.

## Discussion

The objective of this study was to gain some preliminary insight into the IPSJ in male CrossFit participants of different competitive levels. Significant differences were observed in aspects of the IPSJ between the advanced level as compared to the intermediate and beginner level groups. These results complement previous findings for CrossFit participants (Mangine et al., 2020), offering further insight into shoulder muscle performance and injury risk in this athletic population. Indeed, in sports with a high incidence of shoulder injuries, such as handball and artistic gymnastics (Hinds et al., 2019; Moller et al., 2017), the evaluation of the IPSJ, particularly of the external/internal rotation ratio, can provide important information with respect to one’s risk of sustaining a shoulder injury. This is also applicable in sports with a high volume of overhead movements (Ellenbecker & Davies, 2000) such as CrossFit, an activity in which the shoulder is typically one of the most commonly injured body parts (Barranco-Ruiz et al., 2020; Carbone, Candela & Gumina, 2020; Da Costa et al., 2019; Dominski et al., 2018; Gean et al., 2020; Rodríguez et al., 2021; Summitt et al., 2016). Indeed, recent systematic reviews on the prevalence of injuries in exercise programmes based on CrossFit, cross training and high-intensity functional training methodologies (Barranco-Ruiz et al., 2020), as well as other weight-training sports including weightlifting, powerlifting and strongman (Keogh & Winwood, 2017), observed that the body part that was most commonly injured was the shoulder. Accordingly, our findings offer relevant information for CrossFit practitioners and the professionals who assist in their preparation.

One of the primary findings of this study was the higher torque (relative to body mass) of the internal rotator muscles of the shoulder in the advanced level group when compared to the intermediate and beginner levels. This finding is similar to the greater peak torque/body mass at 60°.s^−1^ and 180°.s^−1^ observed in volleyball players from higher competitive divisions, as compared to those in lower divisions (Mendonça et al., 2010). Similarly, it is comparable to the greater peak torque/body mass observed at 60°.s^−1^, 120°.s^−1^ and 180°.s^−1^ in boxers from higher competitive divisions, as compared to those in lower divisions (Tasiopoulos et al., 2018). These differences observed between competitive levels may be related to different training loads and training ages between the levels and could indicate the importance of developing the shoulder internal rotators for these sporting activities. For example, male CrossFit participants competing at the advanced level are often exposed to a greater number of competitive events and training sessions when compared to participants competing at the beginner level (Da Costa et al., 2019). On the other hand, unlike other studies involving volleyball players and boxers (Mendonça et al., 2010; Tasiopoulos et al., 2018), the torque of the external rotator muscles of the shoulder was similar across male CrossFit participants at the different competitive levels, indicating a potentially deleterious imbalance that could increase the risk of injury as the participants progress up the competition levels. The lack of a significant difference in external rotators between levels may be due to the fact that training routines in CrossFit aim to improve strength and physical conditioning in specific patterns of movement (Hak, Hodzovic & Hickey, 2013; Mangine et al., 2020), without considering the balance between the activation or development of agonist/antagonist muscles (Glassman, 2007). For example, the Olympic lifts, as well as kipping/butterfly wide grip pullups, muscle ups and handstand push-ups, commonly practiced in CrossFit (in all competitive levels) require the participant to adopt an extreme position of shoulder abduction, flexion and internal rotation during performance (Hak, Hodzovic & Hickey, 2013). It is also possible that the high number of repetitions of this type of movement could also reinforce any negative outcomes due to this activity. This can translate into greater torque development for the internal rotators compared to the external rotators, representing an imbalance in the way that each is targeted through training. This can be considered as a factor that could explain the higher prevalence of shoulder injuries in CrossFit participants of advanced competitive level as the volume and intensity of their work contributes to the described imbalance (Da Costa et al., 2019). Our findings could have practical applications for CrossFit training in that there may be a need to include supplementary movement routines of shoulder external rotation that complement the strengthening of the internal rotator muscles that occurs as a result of commonly performed upper body exercises.

Our results also revealed greater mean power values in internal rotation and internal diagonal movements of the shoulder in the advanced level group as compared to the intermediate and beginner level groups. Although there is a lack of studies assessing this parameter in athletes from different competitive levels, some investigations have been conducted in tennis (Chandler et al., 1992) and baseball (Newsham et al., 1998), sports in which one limb is used predominantly, and preferentially, over the other. In the aforementioned studies, a greater mean power was observed for the internal rotators of the shoulder in the dominant arm, compared to the non-dominant arm, at 60°.s^−1^ in tennis and at 180°.s^−1^, 300°.s^−1^ and 450°.s^−1^ in baseball. Thus, a greater load seems to be imposed on the dominant limb and this could selectively induce a higher level of power in the internal rotators given the kinematic characteristics (*e.g*. high speed) of the movements performed in both tennis and baseball. It should be noted that the exercises derived from Olympic lifting, as well as the kipping/butterfly pull up, muscle up and push-up derivatives practiced in CrossFit, involve significant bilateral power development (Glassman, 2007; Hak, Hodzovic & Hickey, 2013), differentiating those exercises from what has been described in sports with a unilateral upper limb component. Indeed, unlike sports such as tennis (Chandler et al., 1992) and baseball (Newsham et al., 1998), our results revealed symmetrical power values between the right and left shoulders. The greater mean power values in the advanced level group might be associated with the higher training loads undertaken by that cohort compared to the intermediate and beginner participants, although this needs further confirmation Furthermore, our findings suggest that isokinetic muscle power could help to characterise differences between competitive levels among male CrossFit training participants.

Regarding the peak torque ratio between external-internal rotators, the advanced level group presented a lower ratio in the rotational pattern at 300°.s^−1^ and 180°.s^−1^, compared to the intermediate level group. This finding differs from previous studies in boxers (Tasiopoulos et al., 2018) evaluated at 60°.s^−1^, 120°.s^−1^ and 180°.s^−1^ in whom no significant differences were observed between levels (*i.e*. elite vs amateur). However, in volleyball players of different competitive standards (Mendonça et al., 2010), significant differences were evidenced at 60°.s^−1^ and 360°.s^−1^. The ratio of peak torque between the external and internal rotator muscles is a variable used to represent the degree of balance between the two opposing muscle groups. Therefore, the differences between levels of CrossFit training participants reported in the present study would reflect a muscle imbalance in the advanced level group. In a recent systematic review, external to internal rotator strength ratios <66% were reported to significantly increase the risk of shoulder injury (Berckmans et al., 2017). In the present study, only the advanced level group had values <66% in all analysed movements (*i.e*. rotational; diagonal) and speeds (*i.e*. 180 °.s^−1^, 300 °.s^−1^) and this was more pronounced in the dominant shoulder. We found no significant differences in external rotator torque between competitive levels but there were differences between levels for the internal rotators, with these being larger in the advanced level group. Accordingly, external-internal rotator ratios <66% in the advanced level group are mainly attributed to an imbalance toward greater development of internal rotators compared to the external rotator muscles of the shoulder, a force profile that could result in compression of the subacromial space and inflammation of the surrounding tissue (Page, 2011). Thus, it is possible that CrossFit participants of a relatively advanced competitive level could be considered to be of a greater risk of injury than their lower-ranked/skilled counterparts. Such greater risk has indeed been observed in previous studies (Da Costa et al., 2019; Weisenthal et al., 2014), thus supporting our original hypothesis. On this basis, the peak torque ratio between the external and internal rotators muscles of the shoulder must be considered in the routine assessment of CrossFit training participants, particularly those competing at advanced levels of the sport. Such assessments may help to identify the participants who are at increased risk of sustaining future shoulder injuries, with such participants recommended to increase the frequency, volume and intensity of exercises that develop the shoulder external rotators.

Of note, across all three competitive levels of CrossFit, no significant differences were found between the dominant and non-dominant limbs, for any of the dependent variables analysed. This suggests that CrossFit involves the symmetric development of strength and power for the muscles of the dominant and non-dominant shoulder, possibly indicating that the emergence of asymmetries in this population is less likely. In contrast, significant asymmetries have been reported in sports such as baseball (Newsham et al., 1998), volleyball (Mendonça et al., 2010), boxing (Tasiopoulos et al., 2018) and tennis (Chandler et al., 1992), where there is a strong tendency for the dominant shoulder to be injured as a result of the unilateral nature of these sports (especially baseball, volleyball and tennis).

The primary limitation of this study may be the relatively small sample size though this appeared to be sufficient to obtain 80% statistical power. Another limitation of our study relates to the lack of functional isokinetic indices assessment (*e.g*. shoulder functional proportion of deceleration). Considering that differences have been found between such indices and the ones used in this study (Berckmans et al., 2017), future studies should explore this as it is also applicable to participants engaged in CrossFit training and competition.

## Conclusion

The IPSJ in male CrossFit practitioners showed greater torque and power values in those of an advanced competitive level compared to those with a lower competitive level (*i.e*. intermediate, beginner) for the internal rotation and internal diagonal movements, at both 180°.s^−1^ and 300°.s^−1^. However, such differences between competitive levels were, in general, absent for the external rotation and external diagonal movements. Moreover, participants from the advanced level group exhibited an imbalance of peak torque between the muscles responsible for the external and internal rotational and external and internal diagonal movements of the shoulder (*i.e*. peak torque ratio <66%), particularly in the dominant shoulder. This finding suggests a higher probability of shoulder injury in the advanced level group. A complementary training approach is suggested for male CrossFit training participants, particularly those involved in highly demanding training and competitive activities, to support the development of the muscles responsible for the external rotational and diagonal movement of the shoulder. For example, the addition of multiple horizontal pulling exercises, such as inverted rows, single arm rows, bench pulls and face pulls, would be useful, considering the significant electromyographic activity they induce on the external rotators of the shoulder (Boettcher, Ginn & Cathers, 2009; Cricchio & Frazer, 2011). It is suggested that such complementary training approach could reduce the incidence of injuries at the shoulder level, particularly the dominant shoulder.

## Supplemental Information

10.7717/peerj.11643/supp-1 Supplemental Information 1 Data set describing study outcomes. Each data point describes the isokinetic profile of the shoulder muscles for each of the participants in the study

## Additional Information and Declarations

### Data Availability

The following information was supplied regarding data availability: The raw measurements are available in the Supplemental File.
